# Online Learning is a Rollercoaster: Postsecondary Students With Learning Disabilities Navigate the COVID-19 Pandemic

**DOI:** 10.1177/07319487221090912

**Published:** 2022-05-04

**Authors:** Lauren D. Goegan, Lily Le, Lia M. Daniels

**Affiliations:** 1 University of Manitoba, Winnipeg, Canada

**Keywords:** learning disabilities, postsecondary education, undergraduate, online learning, COVID-19, phenomenology

## Abstract

Most of what researchers know about the challenges students with learning disabilities (LDs) experience during postsecondary education is based on experiences during face-to-face learning on campus. Less is known about challenges students with LD face during learning online—the mode of instruction students had to navigate during the COVID-19 pandemic. Therefore, the purpose of our research was to examine the lived experience of undergraduate students with LD during their first full semester of online instruction as a result of the pandemic. We interviewed six students in Western Canada and used a phenomenological approach to analyze their experiences. Overall, we extracted six main themes from their interviews. Two of these themes, (a) the broad impact of having LD and (b) accommodations during COVID-19, were specific to being a student with LD. The remaining four themes were more generally related to their overall student experience: (c) online learning is different, (d) the role of others, (e) emotional impact, and (f) resilience and perseverance. We discuss these results in terms of recommendations for future research and teaching in online learning environments.

Attending university can be challenging for students diagnosed with a learning disability (LD) at the best of times (e.g., Lightfoot et al., 2018). For example, although the numbers of students with LD have been growing at postsecondary institutions across Canada (Learning Disabilities Association of Ontario [LDAO], 2018; McGregor et al., 2016), they are typically less likely to complete their degree than their peers (Jackson, 2019) and are more likely to suffer when they lack social integration (Goegan & Daniels, 2020). These struggles are based on their experiences in traditional face-to-face, on-campus learning. With the onset of the COVID-19 pandemic, many institutions were forced to shift from traditional in-person learning to online learning leaving students few options but to adapt to the new approach. This shift has not resolved quickly and instead the vast majority of students continue to “attend university” virtually.

## Learning in the COVID-19 Pandemic

Emerging research on the switch to online learning during the COVID-19 pandemic has demonstrated that undergraduate students experienced challenges such as distractions, reduced focus, increased workload, technology difficulties, and lack of academic support (Hussein et al., 2020). In addition, undergraduate students expressed greater dissatisfaction after their courses were moved online (Means & Neisler, 2021). The greatest area of dissatisfaction was how well students thought they were learning and that they struggled with maintaining motivation in their courses (Daniels et al., 2021; Means & Neisler, 2021). On the contrary, students have also expressed benefits during the switch to online learning for reasons such as saving time and money, experiencing increased safety and convenience being at home, and increasing their participation in classes (Hussein et al., 2020). The emerging studies focus on how students’ learning was impacted by the shift to online learning in the start of the COVID-19 pandemic. However, now that learning largely continues in an online format, it is important to investigate if the early-noted challenges and benefits persist and to attend to the experiences of marginalized groups, such as students with LDs who may experience additional challenges.

## Students With LDs

According to the American Psychiatric Association (2013, p. 66), individuals with an LD can experience one or more challenges related to word reading, reading comprehension, spelling, written expression, and/or mathematics. Moreover, these individuals can have impairments when it comes to abilities such as processing speed, memory (e.g., working and long-term memory), attention, executive functions (e.g., planning and decision-making), and social perception or interaction (Learning Disabilities Association of Canada [LDAC], 2015). These challenges can impact student experiences on campus and their potential for success in their postsecondary pursuits. For example, in their review of the literature, Lightfoot et al. (2018) examined the perspectives and experiences of students with LD at postsecondary institutions and found that students with LD experienced numerous challenges including nonsupportive experiences with professors, challenges with receiving accommodations, and social factors impacting their success (e.g., an ability to make friends and interpret social interactions). Moreover, students with LD report spending more hours studying and doing homework than their peers, and rate themselves lower in terms of academic ability (DuPaul et al., 2017).

### Challenges with online learning for students with LD

Although the majority of research exploring postsecondary students with LD focuses on in-person learning, there are some examples linked to online learning. For example, in Lightfoot et al.’s (2018) literature review, only four studies related to the impact of technology on student learning. Nevertheless, the researchers highlighted several advantages and disadvantages to online learning. Benefits included that communication tools such as chatrooms and discussion boards can facilitate learning and that online learning affords flexibility in scheduling and ease of access to information. Conversely, barriers included delays in response from instructors, reliance on written communication, and challenges with accessing extended time accommodations.

More recently, Cataudella et al. (2021) conducted a review of online learning experiences of students with LD, specifically examining the psychological impact on students. Of note, they identify two bodies of literature, first highlights the lack of inclusive accessibility standards, and the second environments that have high levels of inclusion. The review highlights the importance of inclusive environments for reduced student stress and anxiety, positive self-esteem, and overall well-being. Moreover, investigating the online learning experiences of students with LD is important because these students have been found to be less likely to pass online modules or obtain good grades when compared to their non-LD peers (Richardson, 2015), which is particularly relevant to the online learning environment they found themselves in as a result of the COVID-19 pandemic.

### COVID-19 impact on students with LD

Research examining the experiences of students with LD online specifically during the COVID-19 pandemic is beginning to emerge. Research by Gin et al. (2021) found that students with disabilities broadly (of which more than half identified as having a LD) had difficulties accessing accommodations and campus resources, experienced new challenges online, such as issues with test proctoring technology, reduced access to material and information, and video delivery of information was not always accessible. Specifically, the students identified several challenges with accommodations included instructors making assumptions about what accommodations were appropriate and disability resource centers not providing sufficient information about adapting accommodations for online, which resulted in the need for additional self-advocacy. Specifically for students with LD, Zawadka et al. (2021) examined the experiences of students who identified as dyslexic compared to their peers and found that more students with dyslexia experienced higher stress and identified more difficulties with learning such as adjusting to the pace and work based on their learning needs. Nevertheless, more information is needed to understand the experiences of these students.

## Rationale for the Current Study

Before the COVID-19 pandemic, students with LD chose to learn in-person or online. However, public health restrictions in many jurisdictions around the world removed this choice from students as the vast majority of classes were offered only online for undergraduate students. To date, researchers have noted challenges and possible advantages of learning online for students with LD, but these studies do not take into account the additional parameters of learning during a pandemic. With the ongoing struggles with COVID-19 and the possible continuation of online learning, now more than ever, it is essential to better understand the online learning experiences of these students. Therefore, the purpose of this research was to qualitatively explore the experience of undergraduate students with LD during their first full semester of online as a result of the COVID-19 pandemic. Our main research questions were: What are the lived experience of students with LD during the COVID-19 pandemic and online learning? How do students with LD make sense of their online learning experiences?

## Method

Recruitment for this study took place as part of a broader study examining students’ postsecondary experiences with online learning during the COVID-19 pandemic (Goegan & Daniels, in preparation). Current postsecondary students were eligible to participate in the survey that required them to answer Likert-type scale questions about their learning experiences during the Fall 2020 term. Of the 283 students who completed the survey, 54 self-identified as having an LD, and 11 expressed interest in being contacted for an interview. We emailed 10 people who identified as being in undergraduate studies and excluded one graduate student to create a homogeneous group with a shared experience of undergraduate education. Six people responded to the email, confirming their interest in participating.

### Participants

We gave the six participants pseudonyms to ensure anonymity. Brooke is a 21-year-old White woman in her third year of university in Business with an LD related to reading, written expression, and mathematics. Cassidy is a 22-year-old White woman in her fifth year or higher of university in Arts with an LD related to reading. Elise is a 26-year-old White woman in her second year of university in Agricultural Life and Environmental Science (ALES) with an LD related to mathematics. Liam is a 23-year-old White man in his fourth year of university in Psychology with an LD related to mathematics. Quinn is a 26-year-old White woman in her first year of university in Nursing with an LD related to written expression. Roberta is a 35-year-old White woman in her fifth year or higher of university in Native studies and ALES with an LD related to processing.

### Procedures

The researchers collaboratively created a semistructured interview schedule to guide the conversations. Probes were also created and used to encourage participants’ elaboration on their experiences and to deepen responses. The interview questions and probes are provided in Online Appendix A. The research team used their previous experience with designing interview questions for qualitative research, and developed several *what* and *how* open-ended questions to uncover the lived experiences of the students (Alase, 2017). Participants were sent the general interview questions before their scheduled interviews to help them prepare their responses if they desired, however this was not required.

A research assistant (RA) scheduled online interviews using a video communications program with each of the individuals separately. All the interviews were conducted by the same RA to ensure consistency. This RA was chosen for their background in qualitative research, in particular, phenomenology. The interviews were audio- and video-recorded and lasted between 45 and 75 minutes. The RA began each interview by obtaining consent from the participants and answering any questions. At the end of the interview, participants were asked to provide any additional information they thought was important in understanding their experiences. Following the interview, participants were emailed a $10 gift card in thanks for their time.

### Data Analysis

**Figure 1.**
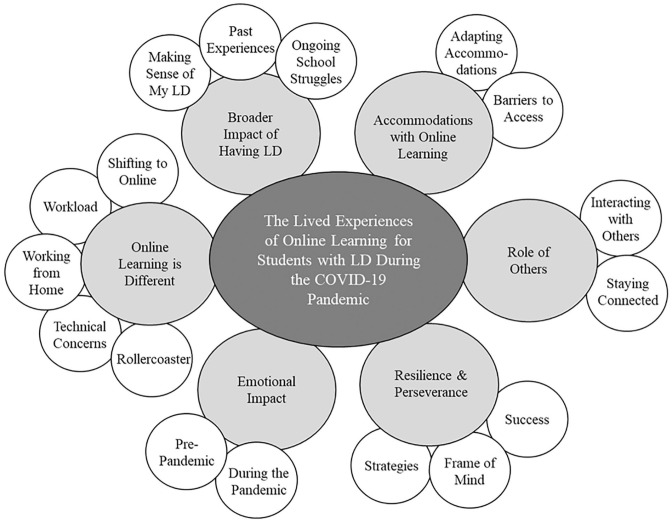
Themes and subthemes from students’ lived experiences.

Phenomenology is the study of phenomena or essences (Finlay, 2009). Researchers do their best to set aside their preconceptions and biases to describe and understand participants’ lived experiences (van Manen, 1990). As such, the researchers reflected on their preconceptions prior to data collection and analysis to reduce bias. The interviews were transcribed verbatim and analyzed by two of the authors following the steps for phenomenological qualitative analysis outlined by Creswell and Poth (2017). First, to obtain a good sense of the data, the researchers reviewed the transcripts several times while memoing emergent ideas in the margins. Second, the researchers developed a “list of significant statements” to understand the participants lived experiences (Creswell, 2013, p. 193). Third, these statements were grouped into broad meaning units (Alase, 2017). Subsequently, we classified the meaning units into themes and subthemes. During this process, the two researchers created a combined codebook after discussing code boundaries and inclusion and exclusion criteria. This included writing descriptions of *what* each meaning unit was and *how* it related to participants’ lived experiences of online learning. The codebook and written descriptions were implemented to ensure inter-rater reliability in interpreting the data and code of the participant statements while eliminating inconsistencies (McAlister et al., 2017). This was an iterative process and involved numerous conversations between the coders and the third author (McAlister et al., 2017). The themes and subthemes are illustrated in Figure 1 and Table 1 which includes corresponding examples. As a form of member checking (Candela, 2019) to ensure credibility of the results, we created a research brief as a verification tool (available upon request) that was sent to participants outlining the themes and subthemes with quotes from the various participants and asked them to comment if these themes and subthemes accurately reflected their experiences (Alase, 2017). All participants responded yes, that the themes were consistent with their lived experiences.

**Table 1.** Themes and Subthemes From Students Lived Experiences With Examples.

Themes and Subthemes	Example
Broad impact of having LD	
Making sense of my LD	“Stigma like you’re dumber than everyone and obviously that’s not true”
Past experiences	“Held back a year”
Ongoing school struggles	“Teach myself everything twice”
Online learning is different	
Shifting to online	“Scrambling to understand how online format worked”
Workload	“They were assigning more extra work”
Working from home structure	“Be able to control my learning environment”
Technical concerns	“The internet connection isn’t well”
Rollercoaster	“It’s been a hit and a miss”
Role of others	
Interacting with others	“Distance and lack of immediacy or flow”
Staying connected	“Relying heavily on my friendly peers in the class”
Accommodations with online learning	
Adapting accommodations to an online setting	“A hurdle I had to overcome”
Barriers to access	“I was really having to fight for it”
Emotional impact	
Prepandemic	**“**I always get overwhelmed”
During the pandemic	“It’s a lot of stress”
Resilience and perseverance	
Strategies	“Organization of both my notes and then my planning of assignments”
Frame of mind	“I’m fairly optimistic”
Success	“But then you figure out how to do it”

*Note.* LD = learning disabilities.

## Results

Based on our interviews, we found the experiences of online learning for students with LD in the Fall 2020 semester were varied. Students described having both positive and negative experiences. We extracted six main themes. Two of these themes (a) broad impact of having LD and (b) accommodations during COVID-19 were specific to students with LD. The remaining four themes were more generally related to their overall student experience: (c) online learning is different, (d) the role of others, (e) emotional impact, and (f) resilience and perseverance.

### The Broad Impact of Having LD

Participants discussed how having an LD impacted their schooling overall, not just during the most recent semester that was impacted by learning during the COVID-19 pandemic. The students reflected on how being a student with LD brought up past experiences in school that tended to be difficult. Some students discussed how they struggled to receive accurate assessments at an early age which left them feeling “disappointed” (Cassidy) with the lack of supports in the classroom. Liam also discussed how he experienced a “tough transition” from high school to postsecondary education as a result of his learning challenges.

As participants discussed how LD shapes their learning experiences, they described how they made sense of their LD. In particular, several students noted the stigma associated with the diagnosis, such as worrying that others may “think you’re stupid” (Brooke) or that “you’re dumber than everyone else” (Cassidy). Having an LD was something that “shaped and influenced spheres of my personality” (Elise) and thus made up a part of the student’s identity.

Moreover, all students described an experience of ongoing school struggle as a student with an LD. These struggles often came in the form of difficulty with learning that required additional work such as having to “teach myself everything twice” (Brooke) and “spend[ing] a lot of time learning through on my own” (Quinn). Learning difficulties also required students to take control of their learning by employing strategies to keep organized, asking questions, and receiving tutoring. The challenges associated with being a student with LD were often described as being different compared to their peers without LD. Quinn describes how she felt like she was in her own world as a student with LD: “You know, you’re off in the corner doing your own thing and it kind of feels like you’re in a totally separate environment from them.”

Furthermore, the experience of being a student with LD was not one the teachers always understood and would sometimes question, raising more obstacles for students. For example,

. . . In high school, I know I struggled . . . like my teachers that were like, “I dunno if you need that extra time” and I’m like, “No I need it.” And they’re like, “No I don’t think I’ll give it to you.” And I’ll have to say, “No, it’s my right.” (Brooke)

The lack of understanding from teachers required students to often self-advocate, sometimes being labeled a “difficult student” (Roberta). Therefore, participants here identified a number of challenges they experienced as students with LD within the larger context of learning.

### Accommodations During COVID-19

All but one participant had accommodations in place when courses were held on campus. These students discussed how accommodations looked differently in an online setting. One student (Quinn) described not using recording devices in asynchronous formats because “I’m right here” (i.e., in her home) and can replay lectures as required. She also mentioned no longer needing the isolation room on campus as a result of working from home. Other students who needed to transfer accommodations online expressed that it was a troublesome process.

In terms of accommodations, you had to register everything before September 30. Which is actually like, you don’t think it’ll be bad cuz you’re like, “I just have to find my exam date.” It’s actually much worse because most of your profs don’t announce the final dates so everything has to be sent to [the name of disability resource centre] because apparently, they can’t do it throughout the semester. I don’t know why but that’s a thing. So that was a hurdle I had to overcome at the beginning of the semester. (Brooke)

Transferring supports that like, I’ve had with [the name of disability resource centre] folks to online has been interesting. So also as much as it’s been a challenge and required a lot of work on my part, I do also feel like I’ve been very well supported. (Elise)

For some students, accommodations were often viewed as necessary to their academic success. They described how both they and instructors were in unchartered territory having to navigate how to adapt their accommodations. In some instances, instructors did their best to accommodate students, such as the few who provided extra time to help students.

It takes like 10 minutes to get into your validation. And so she’s added like 15 minutes at the beginning of the exam, she added extra time thinking that people that don’t have accommodations but might struggle with online exams. So she’s preemptively thought about all these things so it made it so easy. (Roberta)

Nevertheless, some students noted challenges with accessing extended time as one of their accommodations. For example, Brooke reflected on an experience where the instructor reduced the time she was to be allotted: “That 30 minutes drastically changed like how I would do on the exam . . . so I was like stressing.” A similar experience was noted by Cassidy who mentioned having to fight for her accommodation of extra time and that she “know[s] a lot of people that weren’t given the time and a half.” Indeed, Elise also commented on the importance of getting her extend time accommodation, and without it, she would be “crushed.”

Shifting accommodations online also meant that students and instructors had to get creative at times to make it work. Cassidy described how one instructor went above and beyond to accommodate her:

My philosophy teacher, we pick a specific date and a time and she read the exam. She’ll phone me on my phone and we’ll do the exam together on my screen which is super nice. It’s a bit nerve-wracking having your teacher read it but she’s super, super nice and I have no problem with her.

Students also reported experiencing barriers to accessing accommodations. Having to advocate for accommodations was a common occurrence, both when it came to in-person and remote learning settings. The onus to receive accommodations often fell on the students.

Because of my reading issues I sent out an email earlier and I just I’m pretty open so I just say I have a processing disorder and it affects my reading. If I can get the reading list as soon as possible please let me know. Most people are good with it but it’s always the ones that are not good with the reading list that are not good with it and those are the ones that I’m trying to target because when they assign readings like 100 pages to read for the first week I can’t get that done. And I can’t keep up and then I just end up getting more and more behind in class. (Roberta)

And if you didn’t [submit all exam dates] by September 30, you couldn’t register another exam for the whole term. So that means if you forgot one quiz, you wouldn’t get double time for that. So I think that’s really unfair especially since sometimes professors can change something so quickly. (Brooke)

Students occasionally ran into issues with receiving accommodations from professors. Brooke described a time when the professor shortened her allotted time for an exam which led her to have “a mini panic attack” because she realized she would not finish in time. For Cassidy, a professor outright refused her request for accommodation despite her efforts to advocate:

It was just in my Sociology class where I was really having to fight for it. Because all the profs are so worried about us cheating. [. . .] I didn’t choose to have a reading disability right. So it made it a little bit harder. I constantly had to send her emails, I constantly had to get in contact with her to try to figure something out. And we ended up not figuring anything out the day before so I just said, “Screw it” and got a friend to read it for me.”

### Online Learning Is Different

All students described that their courses, which were typically offered in person and on campus, were switched to an online format due to the COVID-19 pandemic. Going from in-person to a remote learning environment was a stressful transition as it involved “scrambling” (Elise) and was a bit of a “learning curve” (Cassidy). This shift brought on several new changes. One change that all participants described was that there was an increased workload in terms of their assignments. These increases in expectations were challenging for students. For example, Roberta said,

I also feel like the workload is a lot more. It’s harder to stay on top of it. Even though it feels like you should be able to do more of it . . . I just find myself just shutting down.

This was similar to Liam’s experience:

Because we weren’t into class they [professors] were assigning more extra work with posting in discussion boards and posting videos of yourself and more reflection and stuff like that. Again, it was a little bit frustrating at the beginning because it seems they were piling on more work to make up for the fact that we weren’t going in.

Students described how working from home was different from in-person classes. On one hand, there were benefits such as cutting down the time and cost associated with transportation. There were also benefits such as “being able to control my own learning environment” (Quinn) and keeping more organized by being in their own space. Brooke mentions:

I don’t know if you study in [library] but it’s like a battle ground, you know trying to find the best table by the window. So you know, not having to worry about that . . . definitely unique about how much I could get more productive. Less of that stress.

On the other hand, working from home raised unique challenges. Students could not control all aspects of their environment, such as when “parents would all of a sudden just walk in the back of your Zoom call” (Brooke) or the physical discomfort and pain having to sit all day. Also, attending school from their homes blurred the boundary between work and home life, resulting in students working more than they typically would. Roberta noted: “There isn’t a separation of church and state anymore . . . the days start to blend, the times don’t seem to have a meaning.” Brooke also commented: “You don’t really know when you can say, this is enough work for the day.”

Students also expressed technical problems such as experiencing poor internet connection, technical feedback, issues with student validation to log in, and concerns regarding whether assignments would successfully be uploaded online. Moreover, some students described that their instructors had their own concerns with remote learning, particularly “assuming that everyone’s gonna cheat” (Cassidy). In the experience of Brooke, instructors were not empathetic when she experienced technical difficulties.

He made a rule that if you started your exam and you had technical issues that were under 15 minutes you wouldn’t get an extra time put on your exam for the time you lost . . .which is really unfair because it’s not someone’s fault that they have these issues. So he said if you had above 15 he would grant you more time. But I dunno, how do you measure something like that? I dunno. So I think that’s a little bit unreasonable.

Overall, many participants acknowledged how shifting to an online learning environment had both positives and negatives. For example, Elise said: “It’s been a bit of a rollercoaster. There’s been a lot of learning and re-learning in terms of how to navigate the academic world online and doing so in a way where you can support yourself.” Moreover, Liam noted,

One good thing is obviously I can stand up and like you know keep myself as focused, do whatever I have to do. So generally speaking, it’s a bit harder to ask questions but at the same time it’s nice to kinda be able to have my own space to learn at the same time. So, there’s a positive and a negative.

### The Role of Others

The role of other people in their most recent semester was also noted by the participants. Students described how interacting with their instructors and classmates looked different compared to in-person classes. They revealed that being physically apart resulted in a disconnect that made learning difficult. Tasks, such as group work were “not conducive to online learning” (Roberta) given that the distance made it “incredibly difficult and really labor-intensive” (Elise). Moreover, attending online classes did not allow many opportunities to connect with classmates, which meant that “the social aspect is pretty much taken out” (Liam).

Online learning also removed the ability to physically connect with instructors. Some students suggested that during in-person classes, they experienced anxiety asking questions, which was partly influenced by their LD. However, some students found that remote learning negatively impacted how they wanted to reach out to their instructors, others stated that they preferred online communication. For example, Quinn noted: “I’m not ever nervous that I’m asking a question in class cuz I’m just typing it in a chat.” Moreover, Liam said:

Personally, I found that I think maybe because of my LD, I preferred not to put my hand up in class. Like if I had a question for example, I would kinda wait until the lecture was done and that way I would kinda just ask my question on the way out and that way I would kinda get a specific answer to what I was asking. So, I found with not being able to talk to the professor after class, it’s a lot harder to kinda get my questions answered.

Also, students described varied experiences in terms of professors’ demonstration of support and understanding, especially in this transition period. Elise describes a mainly positive experience with her instructors:

I’m in four classes this term and I would say three out of four of my profs have been very compassionate and understanding and flexible in kind of what they recognize as achievable for students in terms of workload and deliverables and you know wanting us to stay happy and healthy but also doing the work we need to do.

Another student described the support from instructors as a wide range. Indeed, Brooke said: “It’s like a spectrum . . . your profs heavily dedicated to help you overcome this pandemic. On the other side you have profs who . . . seem overwhelmed and doesn’t know what to do.” Cassidy did not experience the same extent of support from one of her instructors who did not want to provide her with an accommodation for fear of cheating. Cassidy reported that the instructor suggested she find someone to read her the exam as a form of accommodating her LD.

They were like, “Get your family” and I’m like, “My family lives three hours away.” That’s very unrealistic. And I live alone. “Well get your roommate” and I’m like, “I live alone.” Normally even if I had a roommate, I would still get a friend to read it. Like they don’t really understand how uncomfortable it is.

During Fall 2020 semester, students discussed how they experienced increased isolation as a result of the pandemic and remote learning. Although they found comfort in knowing “I am not alone in feeling this way” (Elise) and that “We’re all going through it together” (Quinn), the isolation was difficult. One of the essential aspects of maintaining well-being was connecting with supportive others, such as family, friends, and mental health professionals. For example, Roberta commented, “I’ve actually been relying heavily on my friendly peers in the class—the cohort that I’ve met and I’m very lucky that I’ve been at school and met really supportive friends.” Elise also notes,

. . . there is an emphasis on communication and you know, as far as my friends, we make more of an effort to check in with each other. We’re having pretty honest conversations and you know, there’s a sense of comradery.

### Emotional Impact

In discussing their experiences, students spoke about the stress, anxiety, and mental health issues that were prevalent. For some students, their baseline for stress was already high. Students described tending to be the type of person to “overly stress myself out when it comes to school” (Cassidy) or not seeing stress as something “new for me” (Quinn). Brooke commented:

. . . whenever I start my fall semester at university, I have like a mini panic attack within the first week because it’s just—I always get overwhelmed and I think a lot of it is I get very insecure. And I know in my first year of university I almost dropped out because I was like, “I don’t . . . I’m not gonna survive. Not with my—I have my LD along with, “Why am I here?” My marks aren’t that good. Like, it’s a lot of you get really freaked out so I had that to deal with in my first remote semester.

Some students commented on experiencing additional mental health and life concerns as a result of the COVID-19 pandemic. In particular, the students commented on the emotional impact they experience at the beginning of the semester. Starting the new semester online, “it’s a lot of stress” (Brooke) for students, leaving them feeling “a little frustrat[ed]” (Liam) at having to adjust to a new learning environment online. From the anxiety and stress she felt at the beginning of the semester, Cassidy noted “I psyched myself out at the beginning of the semester I think.” Elise also summaries the start of her academic term as overwhelming:

Yeah in the first two weeks of class I was really really anxious and really overwhelmed by kind of I guess what my perception of online school was gonna be . . . I felt very worked up for a few weeks before classes started being worried about what it was all gonna be like. But those feelings of anxiety and overwhelm really impacted my first like, the first two months of school where I had to get in the flow of things.

Taken together, the impact of the pandemic on students’ education at times exacerbated their pre-existing experiences with mental health and life concerns. Elise noted: “There were a number of other factors at play too, but I ended up having kind of like, a nervous breakdown I guess.” The added impacted of the pandemic “enhances anxiety” (Roberta) and Roberta found herself “shutting down,” while Quinn noted, “it’s kind of exhausting” Moreover, Cassidy said:

I had kind of issues during the summer which didn’t help either. Like I never had depression or mental health issues but it obviously skyrocketed like a lot of people had during this pandemic. So, it kind of just added to my stress level. I had a lot of breakdowns.

### Resilience and Perseverance

Despite the stress and anxiety that students experienced, they were able to remain resilient and persevere through the semester. One way they were able to persist was by employing strategies to help with their learning such as keeping organized, starting and handing assignments in early, keeping track of their schedule, managing their time, and tailoring past learning strategies to their current situation. Tapping into nonacademic strategies such as leaning on social supports, turning to their faith, and engaging in self-care were also essential.

Moreover, students possessed positive beliefs and attitudes that helped them persevere. Students often saw themselves as having resilient characteristics, such as being “fairly optimistic” (Quinn), not having regrets “because I grow and learn from it” (Cassidy) and knowing that “I am capable” (Elise). Even in times where they had little or no control over the situation, they accepted or rolled with it, often with a sense of determination that they could conquer it. Liam said, “It was a little hard to just, you know, swallow the truth of it has to be online now. So that was kind of a bit of an obstacle to overcome.” Roberta commented: “I’ve been in training for this my whole life . . . You kind of feel like the world’s going crazy around you but you’re just like, “OK, What’s next?” Brooke also reflected:

Part of it is keeping a tough mentality about it. I think that’s like part of having an LD. You realize, you know, your educators are not always gonna support you the way you want them to support you so you have to learn to support yourself and give yourself the strategies you need. Essentially to survive whatever situation you’re in.

Students also reflected on their successes over the last semester. Despite remote learning, students identified positive outcomes such as “doing OK gradeswise,” (Liam), “feeling competent,” (Quinn), and “feeling accomplished and happy,” (Cassidy). Also, several students were surprised by their own ability to persist and realized that things turned out better than they initially expected. This reflection was sometimes met with a sense of pride. Cassidy said, “I’ve been getting 80’s, 90’s, 100’s on things so I’m actually quite shocked with my marks that I’m doing really, really well.” Brooke also commented “I always get overwhelmed and like, worried that I won’t finish something. But then you figure out how to do it.” Elise noted, “. . . Reflecting back on the last few months, kind of just feeling a sense of accomplishment or pride, or like, really a lot of gratitude and appreciation for how far I’ve come.”

## Discussion

The COVID-19 pandemic provided an opportunity to examine the online learning experiences of students with LD. Six undergraduate students were interviewed about their experiences online during the Fall 2020 term. The study findings highlighted six themes, two of which were specific to their experiences as students with LD and four of which were more broadly related to their experience with online learning. In this discussion, we consider the interconnectedness between these themes and relate them to existing theory and research. We also discuss the research limitations and make recommendations for potential future research.

### Accessing Accommodations for Students With LD Online

Students with LD described experiencing barriers to accessing accommodations and needing to adapt accommodations to the online setting. While students with LD often experience challenges with accessing accommodations at postsecondary institutions (e.g., Lindstrom, 2007), during the COVID-19 pandemic, these challenges appear to be amplified for some students. Indeed, some participants interviewed identified the process of accessing accommodations as labor intensive and often difficult to obtain thereby adding to an increased workload experienced by these students as discussed below. To support students in accessing accommodations at the institutional level, disability resource centers need to provide clear guidelines as to what students should provide to access their accommodations, as well as transparent timelines for when this information is required (Harrison et al., 2008). This is particularly important when changes are required swiftly, so that students with LD who experience challenges with executive functioning such as planning and working memory are able to adapt successfully and access the appropriate supports.

The transformation of accommodations to fit an online setting was met with both positives and negatives. Some students mentioned certain accommodations were not necessary for online learning. For example, students who recorded lectures when in-person did not require this accommodation for asynchronous classes. Moreover, writing exams in an isolated space was not necessary, or possible, when working from home, given the parameters of the student’s home working environment. Therefore, it would be advantageous for students to review their accommodation needs prior to registering in online courses, when possible, to ensure they can be properly supported. On the contrary, students also noted some accommodations were difficult to obtain, and some instructors were more accommodating than others. This challenge with some instructors was also mentioned as a broader impact of having LD, perhaps signifying an ongoing challenge in the education system for students with LD. Therefore, additional information should be provided to postsecondary instructors about the necessity of accommodations for certain students and how best to incorporate suitable accommodations into their courses.

One accommodation that was mentioned consistently by students was extended time. Extended time is the most common accommodation requested by students with LD, and it has several challenges associated with it as well (Goegan & Harrison, 2017). The challenges of obtaining extended time may be exacerbated during online learning as there is often an increased concern with cheating (Daniels et al., 2021). Nevertheless, instructors need to ensure that students are provided with their accommodations as outlined in psychoeducational assessments (Harrison et al., 2008). Other times, professors provided more time for all students, acknowledging additional time required with exams online. This approach is in line with universal design for learning (UDL; CAST, 2018), where all students should be provided with appropriate time to complete the task, particularly when time is not a parameter of interest but rather a potential confound to measuring their acquisition of knowledge or skills in a course. A universal design approach when designing online courses should be implemented to support all learners in a more inclusive setting (Dianito et al., 2021; Petretto et al., 2021). As such, future research could explore the time requirements when completing in-person versus online exams to offer recommendations for time parameters specific to these two learning formats.

Participants also described the importance of self-advocacy, a skill that should begin to be developed during the formative school years (Fullarton & Duquette, 2015). One of the students reflected that they had been “training” for this their whole life, as the strategies and skills they developed over the years were fundamental for their academic success during this new online learning environment as a result of the COVID-19 pandemic. Indeed, the students referenced several strategies or skills they had previously acquired, such as time management skills, organization, taking breaks, and taking time for self-care. These students were able to tailor their learning strategies to this new learning environment to aid their success. While the students here seemed successful in the adjustment of their strategies, this could be an important avenue where other students might need additional support during a transition to a new learning environment. Therefore, educators or postsecondary intuitions might look to providing additional learning strategies for students that are designed for learning remotely.

### Online Learning Is a Rollercoaster

Several students mentioned that online learning was a rollercoaster, and this metaphor seems aptly aligned with how they describe their experiences during the COVID-19 pandemic. The students provided a mixture of ups and down they underwent and provide a balanced view of their learning experiences, consistent with previous research examining students with LD (Petretto et al., 2021) and students more broadly (e.g., Hussein et al., 2020). As such, in the paragraphs to follow, we make recommendations for supports students more broadly emphasizing a universal design approach with online learning during the pandemic, while also highlighting key components for students with LD.

One important area where students identified positives and negatives was the psychological shift to online learning. Our participants expressed the need to find their footing, while also acknowledging that generally speaking, the experience was not as bad as they expected. The fear of the unknown, a fundamental fear (Carleton, 2016), might explain their experiences here. Beyond academics, individuals were facing daily anxiety stemming from the pandemic and the uncertainty that it brings (Kennedy & Boyle, 2021). Much like a rollercoaster at an amusement park, the initial stages of a course exclusively online might have been scary or worrying, resulting in a learning curve, but as it progressed, students highlighted some positives of online learning and their successes in the semester. These included being able to keep pace with others, easily accessible content, and having an increased level of focus in this learning format. As such, to support students with their online courses, we recommend instructors provide clear guidelines and procedures for all their students that are easily accessible. For example, Rao et al. (2021) provide several instructional strategies to support online learning for students with disabilities in particular, such as creating “at a glance,” step-by-step instructions with screenshots that anticipate student questions and concerns, creating short videos with important information, and providing a “resource bank.” These strategies can support a student’s learning online by removing uncertainty and providing appropriate structure. These strategies would be particularly valuable for students with LD, as our participants note the importance of time management and organization skills for their success with learning online. Indeed, students with LD benefit from detailed tutorial materials when learning how to use new online tools (Li et al., 2021).

Another area that students acknowledged had positives and negatives was the working from home structure. Indeed, some of the students appreciated the flexibility of working from home and not having to commute to school, while others missed the separation of school and home environments. This is consistent with previous work by Hussein et al. (2020), who asked students about the positive and negative aspects of emergency online learning. Effectiveness in terms of time and cost was viewed as a positive aspect, including the reduced time commuting and travel-related costs. Moreover, online learning was seen as convenient. Alternatively, family and personal distractions related to working from home were seen as negative aspects (Zhang et al., 2020). Lack of a proper study space can also be a significant challenge for students (Fidalgo et al., 2020) and was also noted by several of our participants.

Zhang et al. (2020) recommend that to support learning at home, students should try to allocate a certain space in the home for school. This can help students focus when engaging with school materials. Moreover, this designated space should be avoided when not working on school tasks to maintain the separation of school and home that students want. In addition to the physical space, students should be mindful of how they are using their time. For example, developing clear study plans or a schedule for the day can support time management skills (Fidalgo et al., 2020), which students with LD often need additional support with (Kreider et al., 2019). Moreover, students mentioned discomfort with sitting all day; therefore, the schedule should also include breaks which could aid with attention challenges experienced by students with LD (LDAC, 2015). In a typical school day, students would move around between classes, and it is important to continue that habit at home as well. In addition, working all day without breaks may be unproductive according to cognitive load theory (Leppink, 2017) and can hinder a student’s memory when studying. Using smaller segments of information when learning, often referred to as “chunking” can support learners in maintaining the knowledge for both short- and long-term memory (Humphries & Clark, 2021). This may be particularly helpful for students with LD who often experience challenges with memory (Trainin & Swanson, 2005).

Nevertheless, two subthemes highlighted mostly negative experiences of students when learning in an online environment, namely the workload and technical concerns. This is consistent with previous research with students in general, who identified negative aspects of the emergency online learning model, which included four themes: distraction and reduced focus (discussed above), workload, technology, and internet connectivity, and inadequate support (discussed below; Hussein et al., 2020). Workload was a common theme from our participants, and this could extend from the broader impact of having an LD. Indeed, many students with LD take a reduced course load at postsecondary to support their learning needs (LDAO, 2018). For students in general, research by Aristovnik et al. (2020) found that 54.7% of North American students surveyed identified that their workload had become larger or significantly larger as a result of the transition to online learning. Therefore, the workload associated with online learning may be doubly detrimental to students with LD. Indeed, research examining online learning of individuals with LD highlighted a decrease in time available for other activities and personal relationships (Lambert & Dryer, 2018), Therefore, increased workload may limit time available for other important activities which are important to maintain, particularly during a stressful time of a pandemic. As such, students might want to evaluate the number of courses they enroll in online to reduce workload. Alternatively, this is an area for increased professional development when educators are learning to teach online (Kennedy & Boyle, 2021). Technical concerns have been raised in previous research examining online learning more broadly (e.g., Aristovnik et al., 2020; Hussein et al., 2020; Zhang et al., 2020) and for students with disabilities in particular (Dianito et al., 2021). This is an important point of consideration instructors and postsecondary institutions when developing courses online.

### The Importance of Staying Connected

The students commented on the role of others, specifically in terms of their interactions in online courses, and more broadly in terms of staying connected outside of school. While participants were in courses with other students, they often felt disconnected from their peers. This may be related to the concept of “connected but alone” by Sherry Turkle (Turkle, 2012). Online courses as a result of the COVID-19 pandemic, seemed to change the in-class dynamic and the interactions between students, which could lead to challenges with socialization and collaboration. Previous research has found that social integration, that is, feeling connected to those around you, is important for academic success for students general and is particularly important for students with LD and their success at postsecondary (Goegan & Daniels, 2020). Moreover, Lambert and Dryer (2018) suggest that academic and emotional support that is provided to students with LD by family and friends was a key factor in their success with online learning. Indeed, having friends and family as a support system was an important coping strategy for students with disabilities more broadly during online learning as a result of the COVID-19 pandemic (Dianito et al., 2021). As such, educators should be attentive to methods to help students connect with others in the class. For example, engagement online could be enhanced by providing students with opportunities to interact using chat functions, break-out rooms, or incorporating interactive presentation software (Rao et al., 2021). It is important to provide multiple means of engagement as Lightfoot et al. (2018) note a reliance on written communication may pose a challenge for students with LD based on their written language and/or reading impairments (LDAC, 2015). This might also help reduce the stigma these students perceive others to have related to their LD more broadly, if they are able to connect with those around them (Petretto et al., 2021).

Staying connected with others in a general sense is important for the emotional impact of the COVID-19 pandemic. The students interviewed here mentioned numerous emotions such as frustration, stress, and anxiety, which is consistent with previous research examining students with LD in an online environment (Lambert & Dryer, 2018). Students with LD already experience higher amounts of anxiety than their peers without LD (Nelson & Harwood, 2011) and might require additional supports during this particularly stressful time. Staying connected to others thus might be an important strategy for supporting these students. Moreover, relatedness, that is having a sense of belonging and connection to those around you, is one of the basic psychological needs outlined by self-determination theory (Ryan & Deci, 2017). Relatedness is also an important component of the messaging around the COVID-19 pandemic, for example, “we are all in this together” (Porat et al., 2020) and a sentiment that was also expressed by our participants. Therefore, supporting students’ need to enhance their connections with others may be an important component to ensuring a positive experience with online learning.

### Implications for Practice

We highlight two key recommendations for instructors and administrators to support students with LD specifically. This research highlights the importance of keeping students with LD in mind from the outset of preparing for online learning. Rather than treating this group of students as an afterthought in online course design, professional development for instructors should start by considering complex learners and universal design elements (Adam, 2020). One avenue that still requires attention is determining an appropriate workload. Like students without LD in previous research (e.g., Aristovnik et al., 2020), our participants described that learning online in and of itself simply *was* more work, so when the objective workload also increases, the burden may become untenable for students with LD. In a similar vein, institutions and faculty members need to be prepared when it comes to technological requirements for online learning. Although instructors may want to try innovative technologies (Hussein et al., 2020), students may appreciate a simple course structure that can be easily navigated and supported with minimum technology requirements so they can focus on learning rather than technology. This could also reduce potential technical challenges which were often noted as a concern here and with students with disabilities more broadly (Dianito et al., 2021).

In addition to course design, we recommend open and flexible communication between students, faculty, and administrators (Petretto et al., 2021). For example, guidelines from disability resource centers about how to access or adapt accommodations during the pandemic for students could have eliminated some of the stress experienced by our participants. Moreover, greater attention to communication during the COVID-19 pandemic is noted by Petretto et al., 2021, as imperative to support online learning of students with LD. Regular communication from instructors to students may support them in feeling connected, which is critical for their success (Goegan & Daniels, 2020). Staying connected was an important theme found here and in other research examining the lived experiences of students with disabilities more broadly during the COVID-19 pandemic (Dianito et al., 2021), and therefore, requires greater attention.

### Limitations and Future Directions

While our findings here provide important insights into the lived experiences of students with LD during online learning and the COVID-19 pandemic, there are two important limitations that should be noted. First, the sample consists of six undergraduate students from western Canada. How these individuals experienced online learning during the COVID-19 pandemic may have been different from students in other countries that had different guidelines and regulations during the pandemic, or experienced a higher rate of infection and death of citizens, which ultimately could have a stronger emotional impact on students. Moreover, the only indicator of disability status was students’ self-admission. Future research could consider verifying self-reports through registration with disability centers, documentation, and so on. This type of verification, however, can come at the cost of rapport and trust if participants feel the need to prove the veracity of their experiences and is somewhat contrary to the lived experiences being explored in this qualitative study. Nevertheless, the students here provided a descriptive account of their experiences wherein saturation was achieved (Fusch & Ness, 2015), and we have a robust understanding of the experience of the students with LD who were interviewed.

Second, the study was conducted during the Fall of 2020, which represented students’ their first full semester completely online as a result of the COVID-19 pandemic. It could have been advantageous to interview the students again the following semester to see how their experiences were similar or different having already experienced a full 3-month term online. This could be an important avenue for future research as many postsecondary institutions may continue to offer courses online as a result of the pandemic. Alternative, it would have been advantageous to ask students about their previous experiences with courses online to further contextualize the results. Nevertheless, our results provide important information for postsecondary institutions on how to support students with LD with online learning.

### Conclusion

The lived experiences of students with LD with online learning during the COVID-19 pandemic were investigated to examine the challenges and benefits of this learning environment. The responses from the students identified experiences specifically related to being a student with LD such as accessing accommodations online, and the broader impact of having an LD. Moreover, the participants identified several themes related to the student experience more broadly such as how learning online is different, the role of others (e.g., instructors and peers), the emotional impact of learning online during a pandemic and the importance of resilience and perseverance. The themes here provide postsecondary institutions, instructors, and other school personnel with important information for designing online courses for students in the future.

## Supplemental Material

sj-docx-1-ldq-10.1177_07319487221090912 – Supplemental material for Online Learning is a Rollercoaster: Postsecondary Students With Learning Disabilities Navigate the COVID-19 Pandemic Click here for additional data file. Supplemental material, sj-docx-1-ldq-10.1177_07319487221090912 for Online Learning is a Rollercoaster: Postsecondary Students With Learning Disabilities Navigate the COVID-19 Pandemic by Lauren D. Goegan, Ph.D, Lily Le, Ph.D and Lia M. Daniels, Ph.D in Learning Disability Quarterly
