# Supplementary material for: Online Learning is a Rollercoaster: Postsecondary Students With Learning Disabilities Navigate the COVID-19 Pandemic
Source: Learn Disabil Q. 2022 May 4;46(3):166–79. doi: 10.1177/07319487221090912 (PMC10354527; doi:10.1177/07319487221090912)
Supplement: sj-docx-1-ldq-10.1177_07319487221090912 – Supplemental material for Online Learning is a Rollercoaster: Postsecondary Students With Learning Disabilities Navigate the COVID-19 Pandemic [file sj-docx-1-ldq-10.1177_07319487221090912.docx]

**Appendix A**

**Interview Questions and Prompts**

1. Before we talk about your experiences this term, can you walk me through a typical day for you last term?
2. And now can you walk me through a typical day for you this term?
3. What has it been like to be a student with LD **this term**?
   1. What’s it like to be “at” university this term?
   2. What are your experiences at university/college this term?
   3. What are classes like this term?
4. What is **unique** about this semester for your learning? OR What’s different about this term than previous ones?
   1. What is unique about your classes?
   2. What is unique about your Interactions with other students/instructors?
5. What types of **skills/attitude/beliefs** do you think influenced the way you experienced different situations this term? OR What **skills/attitude/beliefs** are helping you this term?
6. How have you **felt** this semester? / What emotions have you **felt** this semester?
   1. What specific experience has made you feel _______?
   2. Positive emotions?
   3. Negative emotions?
7. What **barriers** have you experienced this semester? OR Can you tell me about a specific **struggle** you experienced this term?
   1. What was it like?
   2. How did you feel?
   3. What did you do?
8. How have you been **successful** this term? OR Can you tell me about a specific **success** you experienced this term?
   1. What was it like?
   2. How did you feel?
   3. What did you do?
9. What supports or services have you accessed this term?
   1. How has it been helpful? Not helpful?
10. What else would you like to add about your experience with LD this term?
